# A suicide inhibitor of nematode trehalose-6-phosphate phosphatases

**DOI:** 10.1038/s41598-019-52593-9

**Published:** 2019-11-07

**Authors:** Megan Cross, Mark York, Ewa Długosz, Jan Hendrik Straub, Sonja Biberacher, H. M. P. Dilrukshi Herath, Stephanie A. Logan, Jeong-Sun Kim, Robin B. Gasser, John H. Ryan, Andreas Hofmann

**Affiliations:** 1 Griffith Institute for Drug Discovery, Griffith University, Nathan, Queensland, 4111 Australia; 2 CSIRO Biomedical Manufacturing Program, Clayton, Victoria 3168 Australia; 3 Department of Preclinical Sciences, Warsaw University of Life Sciences, 02-787 Warsaw, Poland; 4 Department of Veterinary Biosciences, Melbourne Veterinary School, The University of Melbourne, Parkville, Victoria 3010 Australia; 5 Department of Chemistry, Chonnam National University, Gwangju, 61186 Republic of Korea; 6 Queensland Tropical Health Alliance, Smithfield, Queensland 4878 Australia

**Keywords:** Chemical libraries, Chemical modification, Enzymes, Mechanism of action, Natural products, Proteins, Screening, Small molecules, Infectious diseases, Antimicrobials, Parasitology, Pathogens

## Abstract

Protein-based drug discovery strategies have the distinct advantage of providing insights into the molecular mechanisms of chemical effectors. Currently, there are no known trehalose-6-phosphate phosphatase (TPP) inhibitors that possess reasonable inhibition constants and chemical scaffolds amenable to convenient modification. In the present study, we subjected recombinant TPPs to a two-tiered screening approach to evaluate several diverse compound groups with respect to their potential as TPP inhibitors. From a total of 5452 compounds tested, *N*-(phenylthio)phthalimide was identified as an inhibitor of nematode TPPs with apparent *K*_i_ values of 1.0 μM and 0.56 μM against the enzymes from the zoonotic roundworms *Ancylostoma ceylanicum* and *Toxocara canis*, respectively. Using site-directed mutagenesis, we demonstrate that this compound acts as a suicide inhibitor that conjugates a strictly conserved cysteine residue in the vicinity of the active site of nematode TPPs. The anthelmintic properties of *N*-(phenylthio)phthalimide were assessed in whole nematode assays using larvae of the ascaroids *T. canis* and *T. cati*, as well as the barber’s pole worm *Haemonchus contortus*. The compound was particularly effective against each of the ascaroids with an IC_50_ value of 9.3 μM in the survival assay of *T. cati* larvae, whereas no bioactivity was observed against *H. contortus*.

## Introduction

Although in the public perception risks from infectious diseases caused by parasitic worms (helminths) of humans, animals and plants are often ranked moderate or low^1^, many of these diseases have a substantial impact in agriculture, the environment and human health. In particular, with currently >1 billion people infected by roundworms (nematodes)^2^ the resultant morbidity surpasses diabetes and lung cancer in disability-adjusted life years^3^. Worm infections of live stock currently cause annual economic losses of 500 million dollars in Australia alone^4^. In the absence of vaccines for the majority of these parasites, their control relies predominantly on the use of anthelmintics, whose wide-spread use has led to drug resistance problems^5,6^. The present anthropogenic trends as well as the globally changing climate has further resulted in the establishment of parasitic organisms in new habitats^7^. Additionally, an increasing importance of zoonosis^8^ is expected, exemplified by the emerging prevalence of roundworms including *Ancylostoma ceylanicum*^9,10^ and *Toxocara canis*^11,12^ and their effects in humans^12–14^. Toxocariasis, caused by *T. canis* (dog roundworm) and *T. cati (cat roundworm)*, is classified as a neglected parasitic infection that is targeted by the US Centers for Disease Control and Prevention (CDC) for urgent public health action, owing to the threat posed by zoonosis^15^. Other nematode infections, such as haemonchiasis, cause substantial economic losses. The livestock nematode *Haemonchus contortus* (barber’s pole worm) colonises the abomasum in cattle, goats and other wild ruminants and can induce anemia and edema as well as other intestinal disturbances^16^. Often, the host will die with major infections. Therefore, the discovery and development of novel therapeutics is an urgent aim to ensure appropriate control measures for helminths in the future.

The increasing availability of genomic, transcriptomic and proteomic datasets of pathogenic organisms opens up avenues for advanced molecular approaches for drug discovery that utilise data mining to identify novel targets^17,18^. One such target identified by combining genome data set mining and literature research is the enzyme trehalose-6-phosphate phosphatase^19,20^, which is a conserved metabolic enzyme of many pathogens, absent from their mammalian hosts, that catalyses the formation of trehalose. This non-reducing disaccharide constitutes an essential metabolite in many micro-organisms but is neither required nor synthesised by vertebrates. Of the five known trehalose biosynthesis pathways, the so-called OtsAB pathway is the most common and employs trehalose-6-phosphate phosphatase (TPP; Enzyme Commission number 3.1.3.12) for the dephosphorylation of trehalose-6-phosphate (T6P), which is synthesised from uridine diphosphate-glucose and glucose-6-phosphate by trehalose-6-phosphate synthase^21^. The observation that TPP knockdown results in lethal phenotypes in the free-living nematode *Caenorhabditis elegans*^22^ and *Mycobacterium tuberculosis*^23^, combined with the high conservation of this biosynthetic enzyme in pathogenic species^19^ has recently focused efforts of target-based drug discovery on pathogen TPPs (reviewed in^24^).

Previous research efforts have targeted the synthesis of carbohydrate-based substrate mimics with first attempts exploring the usability of the sulphate analogue of T6P, trehalose-6-sulphate. While this analogue showed inhibition of TPPs from different nematode and bacterial organisms, the observed inhibition constants were in the range of 0.05–0.3 mM^25,26^. Recent attempts aimed at phosphonic acid analogues of T6P yielded three compounds with IC_50_ values in the high micromolar range (288–1959 μM)^27^. The trehalase inhibitor validamycin A, an antibiotic compound from *Streptomyces hygroscopicus* with topological similarity to trehalose^28^, was reported to inhibit mycobacterial TPPs in the millimolar range (IC_50_ values 12.6–15.0 mM)^27^. Since carbohydrate chemistry is notoriously challenging and requires delicate protection and de-protection steps during the syntheses of the desired target molecules, an alternative synthetic strategy is to replace one of the two carbohydrate units of trehalose with aryl moieties. The best performing inhibitor from a recently published set of aryl-*D*-glucopyranoside-6-sulphate derivatives inhibited nematode and bacterial TPPs with inhibition constants in the range of 5–100 μM^29^.

In 2007, the Queensland Compound Library, now called Compounds Australia, was established as a national resource and dedicated compound management facility to support biomedical research efforts through the consolidation of small molecules into a repository that facilitates subsequent screening^30^. The compound repository includes collections from academic researchers as well as commercially sourced compounds and augments a large variety of drug discovery efforts, including target-based drug discovery programs^31^.

Here, we used compound collections from local academic research groups, the public-private partnership Medicines for Malaria Venture as well as Compounds Australia to evaluate selected diverse compound groups for interactions with pathogen TPPs.

## Results

### Screening of compound libraries against *A. ceylanicum* TPP

Given the exquisite substrate specificity of TPPs^32–35^, there is a strict requirement for the original and rather expensive substrate trehalose-6-phosphate in enzyme assays testing the activity and inhibition of these proteins. Therefore, we employed an economical two-tiered screening approach that consisted of a first-stage screening of compound libraries with a ligand binding assay based on thermal protein denaturation and a second-stage validation of hits in enzyme activity assays^31,36^.

For the ligand binding assay, *A. ceylancium* TPP was used as a target against which four different compound libraries were screened for potential effectors. The chosen libraries comprised purified or re-synthesised natural products from local chemistry groups, the Pathogen Box from Medicines for Malaria Venture (MMV), as well as representative subsets of the CSIRO synthetic library^37^ and the Open Scaffold Collection^38^ available from Compounds Australia at Griffith University (see Table 1). Using a minimum temperature difference of 1.5 K between experiments in the presence and absence of compound as cutoff criterion, 263 hits were identified of a total of 5452 compounds tested, which corresponds to an overall hit rate of 4.8%. All compounds were tested in triplicate, requiring 182 multi-well differential scanning fluorimetry experiments; the mean temperature at the inflection point of the *Acey*-TPP (negative control) unfolding curves was 46.2 °C ± 0.2 °C using a confidence interval of 95%.

**Table 1:** Compound libraries screened in the ligand binding assay.

Library	Origin	Number of compounds	Number of hits (|Δ*T*_m_| > 1.5 K)
Natural products	Local chemistry research groups	531	28
Pathogen Box	Medicines for Malaria Venture, Geneva, Switzerland	400	80
CSIRO synthetic library (representative subset)	CSIRO, Melbourne, Australia	2070	133
Open Scaffolds Library (representative subset)	Compounds Australia, Griffith University	2451	22
	**Total**	5452	263

Of the hits obtained from the ligand binding assay, 222 compounds were tested as competitive inhibitors at a concentration of 25 μM in an endpoint assay of phosphatase activity by *A. ceylanicum* TPP using T6P as the substrate; 41 compounds were not available at the time for further experiments. In this validation step, *N*-(phenylthio)phthalimide (**1**) of the CSIRO library displayed greater than 50% inhibition of *Acey*-TPP enzyme activity compared with the control experiment without inhibitors.

### Evaluation of a phthalimide analogue series against pathogen TPPs

**Figure 1:**
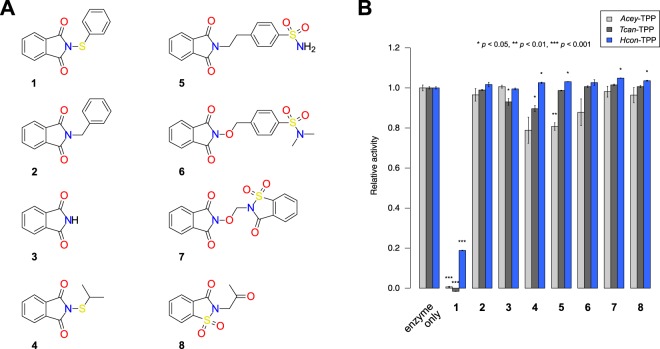
Evaluation of the enzyme inhibition properties of the hit compound and an analogue series. (**A**) Structures of the original hit *N*-(phenylthio)phthalimide (**1**) and a series of readily available analogues (**2**–**8**) investigated in this study. The structures were drawn by the authors using the software cDraw^60^. (**B**) Results of inhibition experiments in the phosphatase enzyme assay of the TPPs from *A. ceylanicum* (light grey), *T. canis* (dark grey) and *H. contortus* (blue) using T6P as substrate and no or 25 μM of compound. Only the original hit compound **1** displayed substantial reduction of the phosphatase activity. Bars indicate the mean relative activity of three independent experiments; error bars indicate the standard error. Statistical significance of the difference between **1**–**8** and the control experiment is indicated using asterisks.

To obtain preliminary information about structure-activity relationships, seven analogues (**2–8**; Fig. 1A) were evaluated in addition to *N*-(phenylthio)phthalimide (**1**) for their inhibitory properties at a concentration of 25 μM in the phosphatase endpoint assays for TPPs from each *A. ceylanicum, T. canis* and *H. contortus* (Fig. 1B). Compared with the uninhibited enzymes, **1** resulted in 82% inhibition of *Hcon*-TPP1 and complete annihilation of *Acey*-TPP and *Tcan*-TPP enzyme activity, respectively. However, neither phthalimide (**3**) nor any of the other six phthalimide analogues elicited any substantial inhibition of nematode TPP enzyme activity beyond 20%.

**Figure 2:**
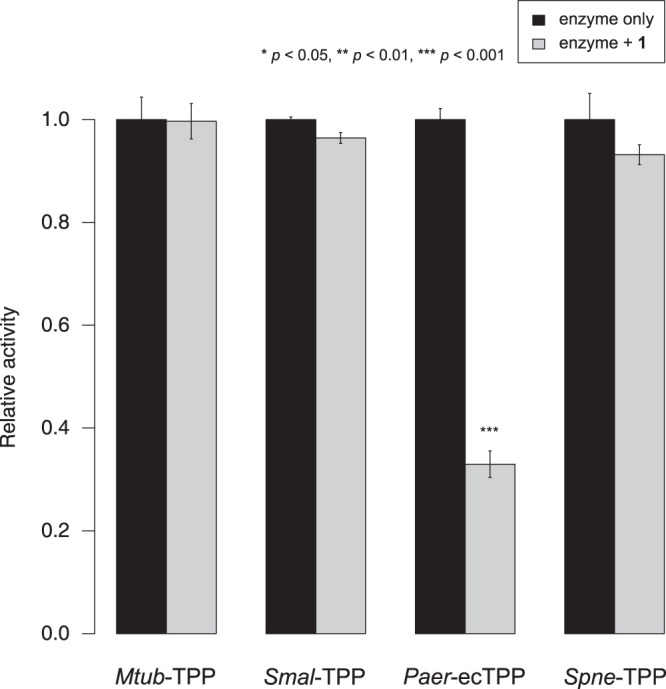
Evaluation of *N*-(phenylthio)phthalimide as inhibitor of bacterial TPPs. *N*-(phenylthio)phthalimide (**1**) was tested at a concentration of 25 μM as competitive inhibitor of the phosphatase activity of the bacterial TPPs from *M. tuberculosis*, *S. maltophilia*, *P. aeruginosa* (extra-chromosomal TPP) and *S. pneumoniae*. Comparison of phosphatase activity in the absence (black) and presence of **1** (grey) revealed a significant reduction of enzymatic activity only for the enzyme from *P. aeruginosa*. Bars indicate the mean relative activity of three independent experiments; error bars indicate the standard error. Statistical significance of the difference between inhibition and control experiment is indicated using asterisks.

**Figure 3:**
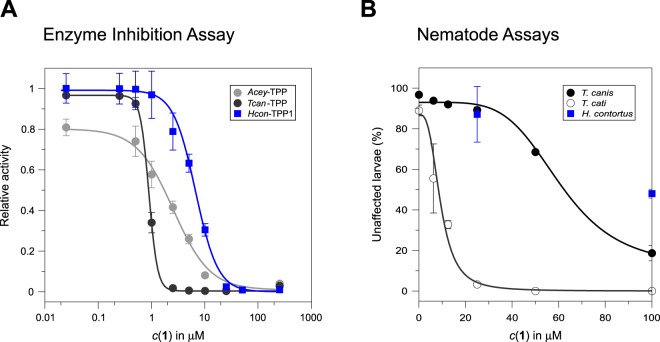
Comparison of dose-response data of *N*-(phenylthio)phthalimide in the phosphatase and nematode assays. (**A**) Dose-response data obtained for *N*-(phenylthio)phthalimide (**1**) in the phosphatase endpoint assay using the TPPs from *A. ceylanicum* (light grey), *T. canis* (dark grey) and *H. contortus* (blue). The enzymatic activity of either enzyme can be almost entirely suppressed by **1** at a concentration >250 μM (*A. ceylanicum*), >25 μM (*H. contortus*) and >5 μM (*T. canis*), respectively. IC_50_ values are summarised in Table 2. (**B**) Dose-response data obtained for **1** (after 24 h exposure) in L3 larvae survival assays indicate half maximum lethal concentrations of 9.3 (±1.4) μM for *T. cati* and 61 μM for *T. canis*. The two data points for *H. contortus* represent motility inhibition at compound concentrations of 25 μM and 100 μM, respectively.

In addition to nematode TPPs, we evaluated the effects of **1** on TPPs from bacteria (*Mycobacterium tuberculosis*, *Stenotrophomonas maltophilia*, *Pseudomonas aeruginosa* and *Streptococcus pneumoniae*) available in our laboratories. Of the four distinct enzymes tested, only the extra-chromosomal TPP from *P. aeruginosa* was susceptible to inhibition by **1** (Fig. 2). The inhibitory effects of **1** on nematode TPPs (Fig. 3A) as well as *Paer*-ecTPP were quantified in dose-response experiments, with apparent inhibition constants in the range of 0.6–2.1 μM (Table 2), assuming a competitive inhibition model.

**Table 2:** Half maximal inhibitory concentrations and derived apparent inhibition constants of *N*-(phenylthio)phthalimide against selected TPPs.

Enzyme	IC_50_ (μM)	app. *K*_i_ (μM)
*Acey*-TPP	2.8 ± 0.29	1.0
*Tcan*-TPP	0.89 ± 0.037	0.56
*Hcon*-TPP1	6.6 ± 0.36	5.8
*Paer*-ecTPP	2.1 ± 0.19	2.1

### Evaluation of redox sensitivity

Since the thioether linkage in **1** might allow involvement of this compound in redox reactions, the effect of different environmental redox states was evaluated. Therefore, phosphatase endpoint assays of *Acey*-TPP and *Tcan*-TPP were repeated in the presence and absence of 1 mM *DL*-1,4-dithiothreitol (DTT) in the reaction buffer as well as in the presence and absence of 25 μM of **1**.

**Figure 4:**
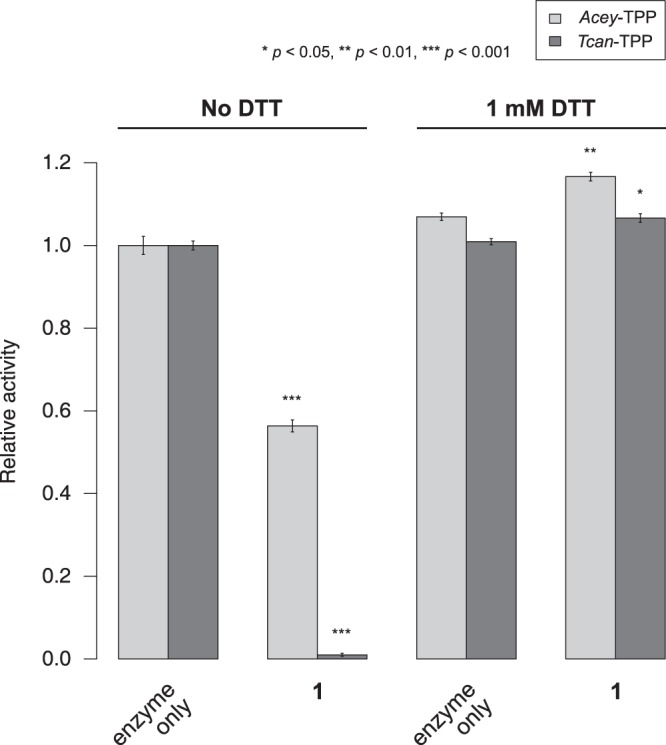
The inhibitory effect of *N*-(phenylthio)phthalimide on the phosphatase activity of nematode TPPs is redox-dependent. *N*-(phenylthio)phthalimide (**1**) was tested at a concentration of 25 μM as competitive inhibitor of the phosphatase activity of the TPPs from *A. ceylanicum* (light grey) and *T. canis* (dark grey) in the absence and presence of 1 mM DTT. The inhibitory effect of **1** was entirely suppressed under reducing conditions. Bars indicate the mean relative activity of three independent experiments; error bars indicate the standard error. Statistical significance of the difference between inhibition and control experiments is indicated using asterisks.

The results demonstrated that the presence of 1 mM DTT did not substantially affect the enzymatic activity of the two nematode enzymes which only showed a marginal increase of activity (*Acey*-TPP: +7%; *Tcan*-TPP: +1%; see Fig. 4). However, the inhibitory effect of **1** on the phosphatase activity of either enzyme was entirely suppressed under reducing conditions.

### Evaluation of the inhibition mechanism

Based on the observed redox sensitivity of TPP inhibition by **1**, we reasoned that a probable inhibition mechanism might involve conjugation of the protein, and we hypothesised that cysteine side chains constitute the most likely conjugation sites. Previously, we derived a topological classification of mono-enzyme TPPs based on a survey of genomes and structure-based amino acid sequence alignments and classified the enzymes into three groups: nematodes, mycobacteria and bacteria^19^. Notably, all members of the nematode TPP group share a strictly conserved cysteine residue situated at the ‘back end’ of the substrate binding pocket at the interface between the cap and the core domain (see Supplementary Fig. S1; *Acey*-TPP: Cys209, *Tcan*-TPP: Cys215, *Bmal*-TPP: Cys222).

Using a molecular dynamics (MD) simulation of a solvated model of *B. malayi* TPP – currently the only nematode TPP with a known experimental three-dimensional structure – we investigated the binding of the substrate trehalose-6-phosphate in the active site. The binding pose obtained from the MD simulation was characterised by a parallel stacking interaction of the phosphorylated glucose unit with Tyr221 (distance ~4 Å; Supplementary Fig. S2) and the phospho group projecting towards the magnesium cofactor. The glucose moiety distal to the phospho group in trehalose-6-phosphate extended into a pocket ‘behind’ Tyr221, and was held in place by hydrogen bonds between the primary alcohol in the 4′-position on the substrate and backbone carbonyl groups of Gly328 (cap domain) and Cys222. Intriguingly, this arrangement suggests that a suitably reactive group of an active site ligand might be able to form a covalent bond with the side chain of Cys222 since the 4′-hydroxyl-O was stably located at a distance of ~3.5 Å from the thiol-S of Cys222 (Supplementary Fig. S3).

**Figure 5:**
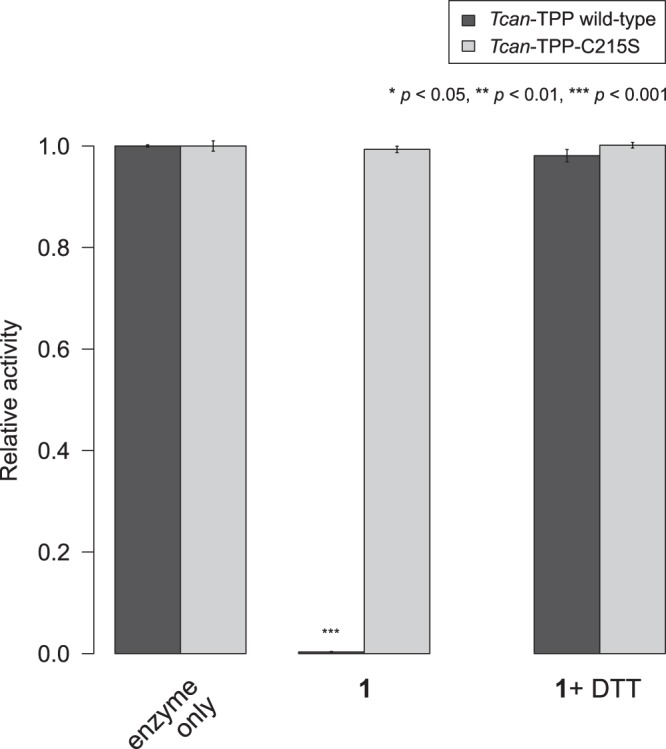
The inhibitory effect of *N*-(phenylthio)phthalimide on the phosphatase activity of *T. canis* TPP requires cysteine 215. *N*-(phenylthio)phthalimide (**1**) was tested at a concentration of 25 μM as competitive inhibitor of the phosphatase activity of wild-type (black) and the C215S mutant (grey) TPP from *T. canis* in the absence and presence of 1 mM DTT. The enzymatic activity of the mutant was not affected by **1**. Bars indicate the mean relative activity of three independent experiments; error bars indicate the standard error. Statistical significance of the difference between inhibition and control experiments is indicated using asterisks.

In order to test whether this conserved cysteine residue was involved in the mechanism of inhibition of **1**, we generated a mutant *T. canis* TPP in which Cys215 was replaced with a serine. As evident in Fig. 5, the presence of compound **1** did not affect the enzymatic activity of the mutant protein *Tcan*-TPP-C215S, indicating that the binding of **1** to the side chain of cysteine 215 was a requirement for the inhibition of enzyme activity. The analysis of wild-type *Tcan*-TPP incubated with compound **1** by mass spectrometry confirmed that Cys215 and Cys415 were indeed conjugated with a thiophenyl moiety (Supplementary Fig. S4).

### Effects of *N*-(phenylthio)phthalimide on nematodes

To evaluate whether *N*-(phenylthio)phthalimide (**1**) also possessed anthelmintic effects, the compound was tested in nematode survival^39^, migration (*T. canis*, *T. cati*)^40^ and motility assays (*Haemonchus contortus*)^41^. Whereas the motility of exsheathed *H. contortus* larvae (xL3) was only weakly impaired at a compound concentrations of 25 μM and 100 μM (Fig. 3B), the exposure of *Toxocara* larvae to 100 μM of compound **1** resulted in >80% lethality after 24 h (*T. canis*) and 3 h (*T. cati*), respectively (see Supplementary Fig. S5). The analysis of dose-response data acquired after 24 h exposure to varying concentrations of **1** resulted in half maximum lethal concentrations of 9.3 μM for *T. cati* and 61 μM for *T. canis*, respectively (Fig. 3B).

## Discussion

### Identification of *N*-(phenylthio)phthalimide as a novel inhibitor of nematode TPP enzyme activity

Owing to the exquisite substrate selectivity of trehalose-6-phosphate phosphatases and the rather costly resourcing of its substrate trehalose-6-phosphate, we designed a two-tiered approach for the high-throughput screening of compounds targeting these enzymes. The screening approach consisted of a ligand binding assay and a subsequent enzyme assay as the validation step for prioritised compounds from the ligand binding assay. Using this approach, a natural products library, the MMV Pathogen Box and representative subsets of two compound libraries available through Compounds Australia were mined for potential inhibitors of *Acey*-TPP.

These efforts resulted in the identification of **1** as a potent inhibitor of the phosphatase activity of TPP from *A. ceylanicum*. Subsequent evaluation of inhibition activity with respect to *T. canis* and *H. contortus* TPPs as well as a panel of four bacterial TPPs revealed that **1** preferentially inhibits the nematode enzymes with apparent inhibition constants of 0.6 μM, 1 μM and 6 μM against *Tcan*-TPP, *Acey*-TPP and *Hcon*-TPP1, respectively. Being one order of magnitude lower than previously reported compounds, these values make **1** the most potent nematode TPP inhibitor at present.

### Mechanism of enzyme inhibition

*N*-(phenylthio)phthalimide (**1**) is a close analogue of the fungicide folpet (*N*-(trichloromethylthio)phthalimide) and its partially saturated derivatives captan (*N*-(trichloromethylthio) tetrahydrophthalimide) and captafol (difolatan; *N*-(tetrachloroethylthio)tetrahydrophthalimide). Previously, it has been shown that these chloroalkyl sulfenamides can be conjugated to free thiols, thereby oxidising the thiol to its disulfide and liberating the phthalimide moiety^42^.

Thus, it is plausible that the thiophenyl group of **1** may be conjugated to a cysteine side chain of the nematode TPPs studied to afford a phenylcysteine disulfide modification of the proteins, presumably via an S_N_2 attack of the thiolate on the sulfenamide^43^. Mass spectrometric investigation of *Tcan*-TPP that had been incubated with **1** confirmed thiophenyl conjugation of the cysteine residues at positions 215 and 415.

Reducing conditions, established by the presence of 1 mM DTT, suppressed the inhibitory effect of **1** on the activity of *Acey*-TPP and *Tcan*-TPP, in agreement with earlier observations made with folpet where modification of glyceraldehyde-3-phosphate dehydrogenase by the fungicide was almost entirely prevented in the presence of either 1 mM DTT or reduced glutathione^44^. The suppressed modification of cysteine side chains in a reducing environment might be due to rapid cleavage of the disulfide reaction product of the conjugation reaction or, indeed, cleavage of the sulfenamide and thus loss of the reactant.

The amino acid sequences of nematode TPPs contain several cysteine residues (*A. ceylanicum*: seven, *T. canis*: eight) of which four are strictly conserved throughout the species examined. Importantly, in our previous studies^19^, we noticed that one of these conserved cysteine residues is located in the interface between the cap and core domains of the haloacid dehydrogenase (HAD) fold (Pfam identifier: PF08282), backing on to the substrate binding site. Computational modelling of the substrate-bound TPP from *B. malayi* indicated that the side chain of this conserved cysteine residue maintained a distance of ~3.5 Å to the non-phosphorylated glucose moiety of T6P and was thus in close proximity to the substrate in the active site (Supplementary Figs S2 and S3). Therefore, we reasoned that, given the similarity between T6P and *N*-(phenylthio)phthalimide in terms of their spatial extents and topologies, the latter might be able to populate the substrate binding site and then react with the conserved cysteine residue under non-reducing redox conditions. The absence of any inhibitory activity by **1** when testing the C215S mutant of *T. canis* TPP, fully supports this hypothesis.

Furthermore, the absence of inhibitory effects of **1** on the activity of most bacterial TPPs is in accordance with the proposed mechanism, as bacterial TPPs lack the conserved residue in the core/cap interface. The only exception within the panel of bacterial TPPs tested here is the enzyme from *Pseudomonas aeruginosa* which was susceptible to inhibition by **1** (Fig. 2) with an apparent *K*_i_ of 2.1 μM (Table 2). The reason for this susceptibility is not entirely clear, but we speculate that the conjugation of one or both cysteine residues specific to *P. aeruginosa* (Cys85, Cys231) might lead to conformational alterations that restrict access to the substrate binding site. In particular, it seems likely that modification of the side chain of Cys85 causes a rearrangement of the residues lining the rather narrow access to the active site^35^.

### Anthelmintic properties of *N*-(phenylthio)phthalimide

In order to assess whether findings from the target-based approach can be translated into therapeutic applications, whole organism assays were conducted using two *Toxocara* species (Ascarididae) and *H. contortus* (Trichostrongylidae). Whereas *Toxocara* larvae were susceptible to *N*-(phenylthio)phthalimide with half maximum lethal concentrations in the micro-molar range, no significant effects were observed in motility assays of *H. contortus* larvae despite the inhibitory activity of this compound on the enzyme activity of *Hcon*-TPP1. At present, the reason for this difference in bioactivity remains unclear. It is possible that genus-specific features might have to be considered and/or a different life cycle stage of *Haemonchus* would need to be evaluated (e.g., by employing an egg hatch assay).

## Conclusions

Here, we outlined the application of a ‘systems’ approach to target-based drug discovery. The characterisation and appraisal of a target protein from a range of different organisms capitalises on the advances of ‘omics disciplines over the past decade and the resultant growing resources in the form of genome and transcriptome datasets. Following selection of trehalose-6-phosphate phosphatase (TPP) as a target of interest and structural appraisal of TPPs from many different pathogenic organisms, these enzymes could be classified into groups^19^ and representatives of each group selected for experimental investigation.

TPPs hold major promise as novel targets of infectious diseases since these enzymes are essential for nematode and mycobacterial pathogens, but are absent from mammalian hosts. Drug discovery strategies for TPPs used thus far have concentrated on the design of substrate mimics, which is challenging due to the fragile behaviour of carbohydrates in chemical syntheses. The clear objective was thus to discover TPP inhibitors with novel chemotypes that provide new avenues for chemical synthetic approaches. Structure-based amino acid sequence analysis of TPPs across different phyla suggested the hypothesis of covalent inhibition of nematode TPPs targeting a nematode-specific cysteine residue in the vicinity of the active site. This hypothesis was tested utilising compound libraries to screen a recombinant nematode TPP and identify novel ligands. The ability of those ligands to inhibit the enzyme activity of TPPs from nematode and bacterial organisms was then evaluated in enzyme assays, revealing that *N*-(phenylthio)phthalimide was a highly effective and specific inhibitor for nematode TPPs. This compound conjugates a strictly conserved cysteine residue in the active site of nematode TPPs and therefore acts as a suicide inhibitor. Whole organism assays with nematode larvae show that *N*-(phenylthio)phthalimide also possesses promising anthelmintic properties against two tested ascaroid species.

These results suggest that TPPs can be worthwhile targets for novel anthelmintics, albeit we can not exclude the possibility that *N*-(phenylthio)phthalimide might also affect other targets in nematodes. Although the particular redox activity of this compound and close analogues may be a hindrance for commercial development, it presents a much needed probe for future studies on TPPs and prof-of-concept for developing covalent, non-substrate-like inhibitors of nematode TPPs.

## Materials and Methods

### Protein expression and purification

The TPPs investigated in this study included the enzymes from the nematodes *A. ceylanicum*, *T. canis* and *H. contortus*; the bacteria *M. tuberculosis*, *Stenotrophomonas maltophilia* and *Streptococcus pneumoniae*; and the extrachromosomal TPP from *P. aeruginosa*^35,45^. All proteins were expressed and purified as over-expressed recombinant proteins according to our previously published protocol^45^. For *H. contortus*, an expression construct in vector p11 (Biodesign Institute, Arizona State University, Tempe, AZ, USA) was generated using the codon-optimised synthetic cDNA (GenScript, Piscataway Township, NJ, USA) of *Hcon*-TPP transcript 1^19^. In brief, the N-terminally hexa-His fusion constructs of all proteins in vector p11 were transformed into *Escherichia coli* BL21-AI cells and 8 litre production cultures in LB+ medium^46^ were seeded from 1 litre of overnight liquid culture. The production culture was grown at 37 °C for 4 h and induced with 1 mM isopropyl β-*D*-1-thiogalactopyranoside (IPTG) and 0.2% arabinose after lowering the temperature to 20 °C. Incubation continued for 40 h, after which cells were harvested, subjected to multiple freeze-thaw cycles, a 5 min sonication and clarification of the cytosolic fraction by high-speed centrifugation. The recombinant proteins were purified by ion exchange and immobilised metal ion affinity chromatography, followed by the removal of the N-terminal hexa-His-fusion peptide by proteolysis with in-house produced tobacco etch virus (TEV) protease. All stages of protein purification were monitored by SDS-PAGE analysis to verify the expected molecular masses for the target proteins.

### Screening of compounds by ligand binding assay

Compound libraries were obtained from local chemistry groups or purchased from Compounds Australia. The libraries were screened in a ligand binding assay against *Acey*-TPP that evaluated the thermal protein denaturation monitored by fluorescence emission of an amphiphilic dye in reaction mixes with and without small molecule compounds (differential scanning fluorimetry; DSF). The optimal ratio of protein and fluorescence dye for a two-state unfolding curve was optimised by testing a 7 × 4 matrix of conditions varying the protein concentration from 0.5 to 32 μM, and SYPRO Orange (Invitrogen; Life Technologies, Mulgrave, VIC, Australia) concentration between 5× and 20× in a sample volume of 20 μL with a buffer composed of 100 mM NaCl and 20 mM HEPES (pH 7.5)^46^. For *Acey*-TPP, the best conditions were determined to contain 0.5 μM protein and 5× SYPRO Orange.

DSF experiments were carried out in 96-well plates using a Roche LightCycler 480 (Roche, Basel, Switzerland). Three technical replicates were tested for each ligand, along with three replicates of a protein-buffer and a protein-DMSO mixture per 96-well plate. Each reaction mixture comprised the optimised protein:dye ratio in a total volume of 20 μL. Ligands were added from their stock solutions in DMSO at a final concentration of 2.5–5 μM, with a final DMSO concentration of 5%. Experiments were analysed using the software DMAN^47^, and Δ*T*_m_ values were calculated as the difference between ligand and DMSO control experiments.

### Phthalimide analogue series

Compounds **1** and **2** were obtained from Combi-Blocks (San Diego, USA) and used as received. For revalidation purposes, compound **1** was also resourced from abcr (Karlsruhe, Germany). Compound **3** was obtained from Sigma-Aldrich (Castle Hill, NSW, Australia). Compounds **4**–**8** were obtained from the CSIRO compound collection.

### Enzyme end-point assays

Trehalose-6-phosphate was synthesised in-house as published previously^19^ and also purchased from Santa Cruz Biotechnology (Dallas, TX, USA). Phosphatase end-point assays were carried out following a previously established protocol^35^. Briefly, the phosphatase activity of *Acey*-TPP and *Tcan*-TPP was assessed using 500 μM trehalose-6-phosphate and 10 μM enzyme to be tested. Reactions were carried out in a volume of 50 μL in assay buffer (100 mM NaCl, 20 mM TRIS, pH 7.5). Individual compounds were added to the reaction mixtures at a final concentration of 25 μM, followed by incubation for 5 min before reactions were initiated by the addition of substrate (final DMSO concentration of 4%).

Reactions were allowed to proceed for 5 min before quenching with 100 μL of BIOMOL^®^ Green reagent (Enzo Life Sciences, New York, NY, USA). Absorbance at 620 nm was determined using a BioTek^®^ Synergy 2 plate reader (BioTek, Winooski, VT, USA) after an incubation period of 15 min for colour development. All reactions were set up in triplicate in 96-well plates (Corning, Sigma-Aldrich, NSW, Australia) at 25 °C and control experiments in the absence of enzyme were used to correct for background absorbance.

Raw data were exported as spreadsheets from the plate reader and assembled for statistical analysis with R^48^ using the software jBar^49^ for calculation of means and standard errors, background correction, normalisation and significance evaluation by a two-sided *t*-test.

To determine IC_50_ values, end-point assays were performed in the presence of increasing concentrations of compound (2.5 nM to 250 μM). Compounds were prepared in stocks of increasing concentration such that only 2 μL was added to reaction wells, thus keeping the DMSO concentration consistent at 4%. Enzyme in the absence of compound (with DMSO only) was run as a control and after correction for background absorbance, all test wells were scaled relative to the enzyme-only control. IC_50_ values were determined through fitting using either the software application SDAR^50^ or DRfit^51^.

### Mass spectrometry

Mass spectrometric analyses were carried out at the Australian Proteome Analysis Facility (APAF) and the following description of methodologies were obtained from the APAF General Report form (A-011 version 3).

To determine possible modification of *Tcan*-TPP by *N*-(phenylthio)phthalimide, the protein was diluted to a final concentration of 1 mg mL^−1^ into a buffer containing 25 μM of **1**, 100 mM NaCl, 0.2 mM MgCl_2_ and 20 mM TRIS (pH 7.5). For in-solution digestion, 20 μL of the protein solution were mixed with 5 μL of 100 mM triethylammonium bicarbonate (TEAB) and 1 μg trypsin was added. After incubation at 37°C for 5.5 h, 2.5 μL of the digested sample were diluted into 7.5 μL of TEAB buffer.

The final sample was subjected to 1D-nano-LC ESI MS/MS analysis using a model 6600 Sciex mass spectrometer and an Eksigent nanoLC-Ultra HPLC system with HALO C18 analytical (160 Å, 2.7 μm, 200 μm × 20 cm) and trap (160 Å, 2.7 μm, 150 μm × 3.5 cm) columns. The loading buffer contained 2% acetonitrile, 97.9% water and 0.1% formic acid; mobile phases A and B consisted of 99.9% water and 0.1% formic acid, and 99.9% acetonitrile and 0.1% formic acid, respectively. The sample (10 μL) was injected onto a reverse-phase trap for pre-concentration and desalted with loading buffer, at 4 μL min^−1^ for 10 min. The peptide trap was then switched into line with the analytical column. Peptides were eluted from the column using a linear solvent gradient from mobile phase A: mobile phase B (98:2) to mobile phase A: mobile phase B (76:24) over 55 min. The reverse phase nano-LC eluent was subjected to positive ion nanoflow electrospray analysis in an information-dependent acquisition mode. A TOF-MS survey scan was acquired (*m*/*z* 350–1500, 0.25 s) with the 20 most intense multiply charged ions (2^+^–5^+^; exceeding 200 counts per second) in the survey scan sequentially subjected to MS/MS analysis. MS/MS spectra were accumulated for 100 ms in the mass range *m*/*z* 100–1800 with rolling collision energy. Dynamic exclusion was set to 30 s.

The sequence of *Tcan*-TPP was added to a database of *E. coli* proteins (23,043 sequences) and the LC-MS/MS data were searched against this database using ProteinPilot v5.0 (SCIEX) in Thorough mode. A custom modification of thiophenyl (C_6_H_5_S, monoisotopic mass: 109.011196 Da) on cysteine was added to the list of modifications.

### Animal ethics

For work with *T. canis* and *T. cati* (Warsaw University of Life Sciences, Poland), no ethics approval was required as no animals were involved in clinical-diagnostic procedures other than requested for their health and with owner permission.

*H. contortus* (Haecon-5 strain) was maintained in experimental sheep (male; 6–8 weeks of age), maintained helminth-free, and housed at the University of Melbourne, as described previously^41,52^. The use of sheep was approved by the Institutional Animal Care and Use Committee of the University of Melbourne (permit no. 1413429). All animal experiments were performed in accordance with the Australian National Health Medical Research council (Australian code of practice for the care and use of animals for scientific purposes, 7th Edition, 2004, ISBN: 1864962658).

### *Toxocara* larvae assays

For the survival assay, an average number of 150 L3 larvae were incubated in 24-well culture plates with serial dilutions of **1** (100 µM–6.25 µM) in Minimal Essential Medium for 24 h at 37 °C, 5% CO_2_. Control larvae were maintained in 0.4% DMSO in Minimal Essential Medium. The survival of L3s exposed to the compound was assessed at several time points after the start of the incubation using a light microscope (at 40× maginification). Larvae were considered alive if they had a characteristic coiled appearance and were motile; they were considered dead if they appeared straight and immobile even after extended observation^39^.

To assess migration, 150 L3 larvae were incubated in different concentrations of **1** (6.25 µM–100 µM). After 24 h of incubation (37 °C, 5% CO_2_), an equivalent volume of 1.5% agar was added to each well^40^. The agar was allowed to set prior to the addition of 0.3 ml of phosphate-buffered saline to each well and the plates were again incubated for 24 h at 37 °C, 5% CO_2_. The number of larvae that migrated to the top of the well was counted using a light microscope (at 40× magnification).

### *H. contortus* larvae assays

The effects of compound **1** were assessed in a motility assay using exsheathed third-stage larvae (xL3s) of *H. contortus* in 96-well microculture plates (Corning 3635; Life Sciences, USA) and the control compounds moxidectin and monepantel, following the published protocol^53^. In brief, **1** was diluted to the final concentration of 100 µM using Luria Bertani medium (LB) supplemented with 100 IU/mL of penicillin, 100 μg mL^−1^ of streptomycin and 2.5 μg mL^−1^ of amphotericin (LB*), and then dispensed in triplicate into wells of a 96-well microculture plate using a multichannel pipette. Additionally, the negative controls (LB*, LB* + 0.5% solvent; six wells each), and positive controls (20 µM monepantel; Zolvix, Novartis Animal Health, Switzerland and 20 µM moxidectin; Cydectin, Virbac, France; triplicate wells) and xL3s (~300 per well) were dispensed into wells of the plate using an automated multichannel pipette. Following incubation at 38 °C and 10% CO_2_ for 72 h, a video recording of 5 s duration was taken of each well using a grayscale camera (Rolera Bolt, QImaging, Canada) and a motorized X-Y axis stage (BioPoint2, Ludl Electronic Products, USA). Videos were processed to calculate a motility index (MI) using an algorithm described previously^41^.

### Modelling of substrate-bound *B. malayi* TPP

As the deposited crystal structures of *Bmal*-TPP (PDB accession codes 4ofz, 5e0o) lacked several residues due to absence of electron density, we generated a model that included residues 63–491 based on the structure deposited as 4ofz. The resultant model was solvated and subjected to a molecular dynamics (MD) simulation of 20 ns to reduce possible bias.

Using the completed model of *Bmal*-TPP, trehalose-6-phosphate was placed in the vicinity of the presumed binding pocket, but without direct interactions of ligand groups with protein amino acid residues. The binding of the substrate was then investigated by an MD simulation of the solvated system for a period of 20 ns. The results showed that the system attained an apparent equilibrium state after ~3 ns (Supplementary Fig. S3). After ~7.5 ns, the ligand had manoeuvred itself into a binding pose that remained stable for the remainder of the simulation period. Both MD simulations (*Bmal*-TPP in water, *Bmal*-TPP:T6P in water) followed a protocol reported earlier^35^ and were carried out with Gromacs 4.6.5^54^ using the G43a1 force field and the spc water model; the topology for T6P was calculated using the PRODRG2 server^55^. The ligand was manually placed in the binding site with the phospho group projecting away from the magnesium cofactor. Sodium and chloride ions were added by replacing solvent molecules at sites of high electrostatic potential to ensure a charge-neutral cell and at a concentration of 100 mM. For all simulations, periodic boundary conditions were applied in all three dimensions. Following an energy minimisation step, a position-restrained dynamics simulation of 20 ps with a time step of 2 fs was performed to equilibrate the solvated protein complex and gradually equilibrate the system at 300 K and 1 bar. In order to model long-range interactions, the particle mesh Ewald method^56^ was employed with a grid spacing of 1.2 Å. Short-range electrostatic interactions were calculated with a 10 Å cutoff, and for van der Waals interactions a cutoff of 14 Å was used. Temperature and pressure in the simulation cell were controlled with the V-rescale thermostat^57^ and the Parrinello-Rahman barostat^58^, respectively. All bonds were constrained using the LINCS algorithm^59^. The final MD simulation was performed for 20 ns with a time step of 1 fs. Simulations were performed on a custom-built server with an Intel Xeon E5-1650 Six Core (3.5 GHz) and 32 GB RAM. Analyses were performed with Gromacs tools and plots were generated with Grace (http://plasma-gate.weizmann.ac.il/Grace/).

## Supplementary information

Supplementary Information
