# Supplementary material for: A suicide inhibitor of nematode trehalose-6-phosphate phosphatases
Source: Sci Rep. 2019 Nov 7;9:16165. doi: 10.1038/s41598-019-52593-9 (PMC6838324; doi:10.1038/s41598-019-52593-9)
Supplement: Supplementary file 1 — Supplementary Information [file 41598_2019_52593_MOESM1_ESM.pdf]

Supplementary Information

SUPPLEMENTARY METHODOLOGY

Protein expression and purification

In brief, the N-terminally hexa-His fusion constructs of all proteins in vector p11 were transformed into *Escherichia coli* BL21-AI cells and 8 litre production cultures in LB+ medium[1] were seeded from 1 litre of overnight liquid culture. The production culture was grown at 37 °C for 4 h and induced with 1 mM IPTG and 0.2% arabinose after lowering the temperature to 20 °C. Incubation continued for 40 h, after which cells were harvested, subjected to multiple freeze-thaw cycles, a 5 min sonication and clarification of the cytosolic fraction by high-speed centrifugation. The recombinant proteins were purified by ion exchange and immobilised metal ion affinity chromatography, followed by the removal of the N-terminal hexa-His-fusion peptide by proteolysis with in-house produced TEV protease. All stages of protein purification were monitored by SDS-PAGE analysis to verify the expected molecular masses for the target proteins.

Compound characterisation

Compounds were stored by Compounds Australia (Griffith University, Nathan, Queensland, Australia) under robust environmental conditions and supplied in assay ready plate format.

^{1}H- and ^{13}C-NMR experiments were carried out using a Bruker Advance 400 MHz NMR spectrometer at 400 MHz and 100 MHz, respectively. NMR experiments were performed with the sample held at 25±0.1 °C. Chemical shifts for all experiments are referenced using the Unified Scale relative to 0.3% tetramethylsilane in $CDCl_3$ or DMSO-d_6.

High resolution mass spectrometric (HRMS) analyses were carried out using a Thermo Scientific Q Exactive mass spectrometer fitted with an atmospheric solids analysis probe (ASAP®) ion source (M&M Mass Spec Consulting LLC, Harbeson, Delaware, USA); its design and the method of ionisation – a variation of atmospheric pressure chemical ionisation (APCI) – have been described previously[2,3]. The probe was used without flow of solvent, and the nitrogen nebulising/desolvation gas used for vaporisation was heated to 350 °C. The sheath gas flow rate was set to 25, the auxiliary gas flow rate to 5 and the sweep gas flow rate to 2 (all arbitrary units). The discharge current was 4 mA and the capillary temperature was 320 °C. Positive and negative ions were recorded in an appropriate mass range at a mass resolution of 140,000.

Liquid chromatography coupled mass spectrometric (LCMS) analyses were performed using a Waters Acquity UPLC i-Class with QDa mass detector and an adjustment-free atmospheric pressure ionisation (API) electrospray (ES) interface for reliability. Positive and negative ions were recorded simultaneously with full scan analysis in the *m*/*z* range between 100 and 1000. High purity nitrogen (>95%) nebulising/desolvation gas was used for vaporisation with the pressure regulated at 650 – 700 kPa. The probe temperature was set at 600 °C, the source temperature at 120 °C; the cone voltage was 10 V and the capillary voltage was 0.8 kV for both positive and negative ion detection. The chromatographic conditions were as follows:

- Column: Acquity UPLC BEH C18 (50 × 2.1 mm, 1.7 µm particle size)
- Mobile Phase A: 100% Milli-Q Water with 0.1% formic acid

- Mobile Phase B: 100% acetonitrile with 0.1% formic acid
- Flow Rate: 0.400 ml min^{-1}
- Column temperature: 30 °C
- Sample injection volume: 1 µL

The elution protocol consisted of a gradient formed from 95% A to 100% B over 4.50 min; then hold at 100% B for 1 min; change to 95% A over 0.5 min; then hold for 1 min. The MS was collecting data for the complete 7 min run. Spectral absorbance data were acquired in the wavelength range from 190 nm to 350 nm with chromatograms constructed using the absorbance at 254 nm.

Screening of compounds by ligand binding assay

The optimal ratio of protein and fluorescence dye for a two-state unfolding curve was optimised by testing a 7×4 matrix of conditions varying the protein concentration from 0.5 to 32 µM, and SYPRO Orange (Invitrogen; Life Technologies, Mulgrave, VIC, Australia) concentration between 5× and 20× in a sample volume of 20 µL with a buffer composed of 100 mM NaCl and 20 mM HEPES (pH 7.5)[1]. For *Acey*-TPP, the best conditions were determined to contain 0.5 µM protein and 5× SYPRO Orange.

DSF experiments were carried out in 96-well plates using a Roche LightCycler 480 (Roche, Basel, Switzerland). Three technical replicates were tested for each ligand, along with three replicates of a protein-buffer and a protein-DMSO mixture per 96-well plate. Each reaction mixture comprised the optimised protein:dye ratio in a total volume of 20 µL. Ligands were added from their stock solutions in DMSO at a final concentration of 2.5–5 µM, with a final DMSO concentration of 5%.

Enzyme end-point assays

Briefly, the phosphatase activity of *Acey*-TPP and *Tcan*-TPP was assessed using 500 µM trehalose-6-phosphate and 10 µM enzyme to be tested. Reactions were carried out in a volume of 50 µL in assay buffer (100 mM NaCl, 20 mM TRIS, pH 7.5). Individual compounds were added to the reaction mixtures at a final concentration of 25 µM, followed by incubation for 5 min before reactions were initiated by the addition of substrate (final DMSO concentration of 4%).

Reactions were allowed to proceed for 5 min before quenching with 100 µL of BIOMOL® Green reagent (Enzo Life Sciences, New York, NY, USA). Absorbance at 620 nm was determined using a Biotek® Synergy 2 plate reader (BioTek, Winooski, VT, USA) after an incubation period of 15 min for colour development. All reactions were set up in triplicate in 96-well plates (Corning, Sigma-Aldrich, NSW, Australia) at 25 °C and control experiments in the absence of enzyme were used to correct for background absorbance.

To determine IC_{50} values, end-point assays were performed in the presence of increasing concentrations of compound (2.5 nM to 250 µM). Compounds were prepared in stocks of increasing concentration such that only 2 µL was added to reaction wells, thus keeping the DMSO concentration consistent at 4%. Enzyme in the absence of compound (with DMSO only) was run as a control and after correction for background absorbance, all test wells were scaled relative to the enzyme-only control.

Mass spectrometry

To determine possible modification of *Tcan*-TPP by *N*-(phenylthio)phthalimide, the protein was diluted to a final concentration of 1 mg mL^{-1} into a buffer containing 25 µM of **1**, 100 mM NaCl, 0.2 mM $MgCl_2$ and 20 mM TRIS (pH 7.5). Mass spectrometric analyses were conducted at the Australian Proteome Analysis Facility (APAF). For in-solution digestion, 20 µL of the protein solution were mixed with 5 µL of 100 mM triethylammonium bicarbonate (TEAB) and 1 µg trypsin was added. After incubation at 37 C for 5.5 h, 2.5 µL of the digested sample were diluted into 7.5 µL of TEAB buffer.

The final sample was subjected to 1D-nano-LC electrospray ionisation (ESI) MS/MS analysis using a model 6600 Sciex mass spectrometer and an Eksigent nanoLC-Ultra HPLC system with HALO C18 analytical (160 Å, 2.7 µm, 200 µm × 20 cm) and trap (160 Å, 2.7 µm, 150 µm × 3.5 cm) columns. The loading buffer contained 2% acetonitrile, 97.9% water and 0.1% formic acid; mobile phases A and B consisted of 99.9% water and 0.1% formic acid, and 99.9% acetonitrile and 0.1% formic acid, respectively. The sample (10 µL) was injected onto a reverse-phase trap for pre-concentration and desalted with loading buffer, at 4 µL min^{-1} for 10 min. The peptide trap was then switched into line with the analytical column. Peptides were eluted from the column using a linear solvent gradient from mobile phase A: mobile phase B (98:2) to mobile phase A: mobile phase B (76:24) over 55 min. The reverse phase nanoLC eluent was subjected to positive ion nanoflow electrospray analysis in an information dependant acquisition mode. A TOF-MS survey scan was acquired (m/z 350-1500, 0.25 second) with the 20 most intense multiply charged ions (2^+–5^+ ; exceeding 200 counts per second) in the survey scan sequentially subjected to MS/MS analysis. MS/MS spectra were accumulated for 100 ms in the mass range *m*/*z* 100–1800 with rolling collision energy. Dynamic exclusion was set to 30 s.

The sequence of *Tcan*-TPP was added to a database of *E. coli* proteins (23,043 sequences) and the LC-MS/MS data were searched against this database using ProteinPilot v5.0 (SCIEX) in 'thorough' mode. A custom modification of thiophenyl (C_6H_5S, monoisotopic mass: 109.011196 Da) on cysteine was added to the list of modifications.

Modelling of substrate-bound *B. malayi* TPP

As the deposited crystal structures of *Bmal*-TPP (PDB accession code 4ofz, 5e0o) lacked several residues due to absence of electron density, we generated a model that included residues 63–491 based on the structure deposited as 4ofz. The resultant model was solvated and subjected to a molecular dynamics (MD) simulation of 20 ns to reduce possible bias.

Using the completed model of *Bmal*-TPP, trehalose-6-phosphate was placed in the vicinity of the presumed binding pocket, but without direct interactions of ligand groups with protein amino acid residues. The binding of the substrate was then investigated by an MD simulation of the solvated system for a period of 20 ns. The results showed that the system attained an apparent equilibrium state after ~3 ns (Supplementary Figure S3). After ~7.5 ns, the ligand had manoeuvred itself into a binding pose that remained stable for the remainder of the simulation period. Both MD simulations (*Bmal*-TPP in water, *Bmal*-TPP:T6P in water) were carried out with Gromacs 4.6.5[4] using the G43a1 force field and the spc water model; the topology for T6P was calculated using the PRODRG2 server[5]. The ligand was manually placed in the binding site with the phospho group projecting away from the magnesium cofactor. Sodium and chloride ions were added by replacing

solvent molecules at sites of high electrostatic potential to ensure a charge-neutral cell and at a concentration of 100 mM. Following an energy minimisation step, a position-restrained dynamics simulation of 20 ps with a time step of 2 fs was performed to equilibrate the solvated protein complex and gradually equilibrate the system at 300 K and 1 bar. Periodic boundary conditions were applied in all three dimensions. Long-range interactions were modelled using the particle mesh Ewald method[6] and a grid spacing of 1.2 Å; the cutoffs for computation of short-range electrostatic and van der Waals interactions were 10 Å and 14 Å, respectively.

The temperature was controlled with the V-rescale thermostat[7] and the pressure with the Parrinello-Rahman barostat[8]. All bonds were constrained using the LINCS algorithm[9]. The final MD simulation was performed for 20 ns with a time step of 1 fs. Simulations were performed on a custom-built server with an Intel Xeon E5-1650 Six Core (3.5 GHz) and 32 GB RAM. Analyses were performed with Gromacs tools and automated plots generated with Grace (http://plasma-gate.weizmann.ac.il/Grace/).

Animal ethics

For work with *T. canis* and *T. cati* (Warsaw University of Life Sciences, Poland), no ethics approval was required as no animals were involved in clinical-diagnostic procedures other than requested for their health and with owner permission. *H. contortus* (Haecon-5 strain) was maintained in experimental sheep (male; 6-8 weeks of age), maintained helminth-free, and housed at the University of Melbourne, as described previously[10,11]. The use of sheep was approved by the Institutional Animal Care and Use Committee of the University of Melbourne (permit no. 1413429). All animal experiments were performed in accordance with the Australian National Health Medical Research council (Australian code of practice for the care and use of animals for scientific purposes, 7th Edition, 2004, ISBN: 1864962658).

***Toxocara* larvae assays**

For the survival assay, an average number of 150 L3 larvae were incubated in 24-well culture plates with serial dilutions of **1** (100 µM–6.25 µM) in Minimal Essential Medium for 24 h at 37 °C, 5% CO_2. Control larvae were maintained in 0.4% DMSO in Minimal Essential Medium. The survival of L3s exposed to the compound was assessed at several time points after the start of the incubation using a light microscope (at 40× magnification). Larvae were considered alive if they had a characteristic coiled appearance and were motile; they were considered dead if they appeared straight and immobile even after extended observation[12].

To assess migration, 150 L3 larvae were incubated in different concentrations of **1** (6.25 µM–100 µM). After 24 h of incubation (37 °C, 5% CO_2), an equivalent volume of 1.5% agar was added to each well[13]. The agar was allowed to set prior to the addition of 0.3 ml of phosphate-buffered saline to each well and the plates were again incubated for 24 h at 37 °C, 5% CO_2. The number of larvae that migrated to the top of the well was counted using a light microscope (at 40× magnification).

***H. contortus* larvae assays**

In brief, **1** was diluted to the final concentration of 100 µM using Luria Bertani medium (LB) supplemented with 100 IU/mL of penicillin, 100 µg mL^{-1} of streptomycin and 2.5 µg mL^{-1} of

amphotericin (LB*), and then dispensed in triplicate into wells of a 96-well microculture plate using a multichannel pipette. Additionally, the negative controls (LB*, LB* + 0.5% solvent; six wells each), and positive controls (20 µM monepantel; Zolvix, Novartis Animal Health, Switzerland and 20 µM moxidectin; Cydectin, Virbac, France; triplicate wells) and xL3s (~300 per well) were dispensed into wells of the plate using an automated multichannel pipette. Following incubation at 38 °C and 10% CO_2 for 72 h, a video recording of 5 s duration was taken of each well using a grayscale camera (Rolera Bolt, QImaging, Canada) and a motorized X-Y axis stage (BioPoint2, Ludl Electronic Products, USA). Videos were processed to calculate a motility index (MI) using an algorithm described previously[11].

SUPPLEMENTARY DATA: COMPOUND CHARACERTISATION

NMR spectroscopic data are reported using the following format: (1) chemical shift (ppm), (2) multiplicity (s = singlet, d = doublet, t = triplet, m = multiplet, br = broad), (3) *J*-coupling constant (Hz), (4) integration.

<u>2-(phenylthio)isoindoline-1,3-dione</u> (**1**)

$C_{14}H_{10}NO_2S$; C1=CC=C(C=C1)SN2C(=O)C3=CC=CC=C3C2=O. HRMS found $[M+H]^+$ 256.0421, requires $[M+H]^+$ 256.0432. LCMS $[M+H]^+$ 163.03 (41%), 469.47 (54%). Product is not stable under the HPLC conditions utilised, the purity of this compound is supported by ^{1}H NMR and by HRMS. δH ($CDCl_3$) 7.96 – 7.90 (m, 2 H), 7.81 – 7.75 (m, 2 H), 7.63 – 7.57 (m, 2 H), 7.36 – 7.29 (m, 3 H). ^{1}H-NMR spectral data were in agreement with those previously published[14].

<u>2-benzylisoindoline-1,3-dione</u> (**2**)

$C_{15}H_{11}NO_2$; C1=CC=C(C=C1)CN2C(=O)C3=CC=CC=C3C2=O. LCMS $[M+H]^+$ 238.14 (98 %). δH ($CDCl_3$) 7.87 – 7.81 (m, 2 H), 7.73 – 7.67 (m, 2 H), 7.46 – 7.41 (m, 2 H), 7.35 – 7.23 (m, 3 H), 4.85 (s, 2 H). ^{1}H-NMR spectral data were in agreement with those previously published[15].

<u>isoindoline-1,3-dione</u> (**3**)

$C_8H_5NO_2$; C1=CC=C2C(=C1)C(=O)NC2=O, LCMS $[M+H]^+$ 148.13 (98 %). δH (DMSO-d_6) 11.33 (s, 1 H), 7.83 (s, 4 H). ^{1}H-NMR spectral data were in agreement with those previously published[16].

<u>2-(isopropylthio)isoindoline-1,3-dione</u> (**4**)

$C_{11}H_{11}NO_2S$; CC(C)SN1C(=O)C2=CC=CC=C2C1=O. LCMS $[M+H]^+$ 222.06 (85 %). δH ($CDCl_3$) 7.97 – 7.91 (m, 2 H), 7.81 – 7.75 (m, 2 H), 3.46 (septet, *J* 6.72, 1 H), 1.26 (d, *J* 6.72, 6 H). ^{1}H-NMR spectral data were in agreement with those previously published[17].

<u>4-(2-(1,3-dioxoisoindolin-2-yl)ethyl)benzenesulfonamide</u> (**5**)

$C_{16}H_{14}N_2O_4S$; C1=CC=C2C(=C1)C(=O)N(CCC3=CC=C(C=C3)S(=O)(=O)N)C2=O. LCMS $[M+H]^+$ 331.01 (85 %). δH (DMSO-d_6) 7.88 – 7.80 (m, 4 H), 7.73 – 7.68 (m, 2 H), 7.43 – 7.37 (m, 2 H), 7.29 (bs, 2 H), 3.85 (t, *J* 7.02, 2 H), 3.01 (t, *J* 7.21, 2 H). ^{1}H-NMR spectral data were in agreement with those previously published[18].

<u>4-(((1,3-dioxoisoindolin-2-yl)oxy)methyl)-*N*,*N*-dimethylbenzenesulfonamide</u> (**6**)

$C_{17}H_{17}N_2O_5S$; CN(C)S(=O)(=O)C1=CC=C(C=C1)CON2C(=O)C3=CC=CC=C3C2=O. HRMS found $[M-H]^+$ 359.0702, requires $[M-H]^+$ 359.0702. LCMS $[M+H]^+$ 361.05 (80 %). δH (DMSO-d_6) 7.88 – 7.85 (m, 4 H), 7.82 – 7.76 (m, 4 H), 5.30 (s, 2 H), 2.60 (s, 6 H). δC (DMSO, 100 MHz) 162.9, 139.3, 135.0, 134.8, 130.1, 128.4, 127.6, 78.1, 37.5.

<u>2-((1,1-dioxido-3-oxobenzo[d]isothiazol-2(3*H*)-yl)methoxy)isoindoline-1,3-dione</u> (**7**)

$C_{16}H_{11}N_2O_6S$; C1=CC=C2C(=C1)C(=O)N(C2=O)OCN3C(=O)C4=CC=CC=C4S3(=O)=O. HRMS found $[M+H]^+$ 359.0342, requires $[M+H]^+$ 359.0338. LCMS $[M-C_8H_4NO_3]^+$ 196.16 (97 %). δH (DMSO-d_6) 8.34 – 8.29 (m, 1 H), 8.24 – 8.19 (m, 1 H), 8.14 – 8.02 (m, 2 H), 7.86 (s, 4 H), 5.76 (s, 2 H). ^{1}H-NMR spectral data was in agreement with those previously published[19].

2-(2-oxopropyl)benzo[d]isothiazol-3(2*H*)-one 1,1-dioxide (**8**)
$C_{10}H_{10}NO_4S$; CC(=O)CN1C(=O)C2=CC=CC=C2S1(=O)=O. HRMS found $[M+H]^+$ 240.0332, requires $[M+H]^+$ 240.0331. LCMS $[fragment]^+$ 214.06 (96%). δH (DMSO-d_6) 8.37 – 8.31 (m, 1 H), 8.16 – 7.99 (m, 3 H), 4.76 (s, 2 H), 2.23 (s, 3 H). ^{1}H-NMR spectral data were in agreement with those previously published[20].

SUPPLEMENTARY FIGURE S1

Structure-based amino acid sequence alignment of nematode TPPs

The alignment was generated with SBAL[21] using secondary structure prediction obtained with PSIPRED[22]. Helical structure is indicated in green, β-strands are shown in red and cysteine residues are highlighted yellow. The domain topology is indicated in the second row. Important amino acid residues mentioned in the main text are annotated with residue numbers based on *B. malayi* TPP.

```
Conserved HAD Motif                             |I |

B. malayi domain           linker |        HAD-core      206-292
                                  213 215 221,222
                                    |  |     ||
>B. malayi            S-----SEAITGKKPIFITDWDGTMKDYCSQYATNL-------------QPVYSA-----   235
>T. canis             E-----SN-TNGRKPIFITDWDGTMKDYCSQYATNL-------------QPVYSA-----   228
>A. ceylanicum        D-----S-AYSGVKPLLVTDWDGTMKDYCSQYATNLQTKLDLVILSESLQPVYSA-----   235
>A. suum              E-----SN-TNGRKPIFITDWDGTMKDYCSQYATNL-------------QPVYSA-----   248
>W. bancrofti         S-----SEAITGKKPIFITDWDGTMKDYCSQYATNL-------------QPVYSA-----   210
>A. simplex           E-----SN-SNGRKPIFITDWDGTMKDYCSQYATNL-------------QPIYSA-----   211
>N. americanus        D-----S-AYSGVKPLLVTDWDGTMKDYCSQYATNL-------------QPVYSA-----   210
>S. ratti             K-----ES-SDGKKPIFVTDWDGTMKDYCSQYATNL-------------QPIYSA-----   195
>D. viviparus         N-----S-AYTGIKPLLVTDWDGTMKDYCSH-----------------------A-----   186
>T. spiralis          ----------TDGLDVFITDWDGTYKTYCCNYRTSV-------------QPAYSA-----   214
>H. contortus_t2      D-----S-ADTGIKPLLVTDWDGTMKDYCSQYATN-------------LQPVYSA-----   191
>H. contortus_t1      D-----S-ADTGIKPLLVTDWDGTMKDYCSQYATN-------------LQPVYSA-----   176
>C. elegans           D-----TSTTGGIKPLFITDWDGTMKDYCSQYATNL-------------QPAYSA-----   214
>C. remanei           D-----TQVT-GKKPLFITDWDGTMKDYCSQYATNL-------------QPAYSA-----   189
>C. brenneri          E-----PHCS-GKKPLFITDWDGTMKDYCSQYATNL-------------QPAYSA-----   175
>C. briggsae          T-----IES-DGIKPLFITDWDGTMKDYCSQYATNL-------------QPAYSA-----   178
>O. dentatum          D-----S-AYSGIKPLLVTDWDGTMKDYCSQYATNL-------------QPVYSA-----   158
```

Sequence accession numbers (gb: GenBank, ps: WormBase ParaSite (PRJEB506), up: UniProtKB): *B. malayi* gb:XP_001893209.1; *T. canis* gb:KHN76157.1; *A. ceylanicum* see[23]; *A. suum* up:F1L1P1; *W. bancrofti* gb:EJW87102.1; *A. simplex* gb:AHY24646.1; *N. americanus* gb:XP_013303829.1; *S. ratti* gb:XP_024500530.1; *D. viviparus* gb:KJH41349.1; *T. spiralis* gb:XP_003379441.1; *H. contortus* (t2) ps:HCOI01636400.t2; *H. contortus* (t1) ps:HCOI01636400.t1; *C. elegans* gb:CAB17072; *C. remanei* gb:XP_003106242; *C. brenneri* gb:EGT40486; *C. briggsae* gb:XP_002645945; *O. dentatum* gb:KHJ98483.

SUPPLEMENTARY FIGURE S2

The binding pose of trehalose-6-phosphate bound to *B. malayi* TPP suggests spatial closeness of substrate and cysteine 222

The figure shows a cartoon drawing of *B. malayi* TPP and the conformation of the bound substrate T6P obtained by molecular dynamics simulation after 20 ns. For the MD simulation of the complex, the deposited structure of *Bmal*-TPP (PDB accession number 4ofz) was completed by adding missing residues and the resultant model was solvated and equilibrated in a separate MD simulation (see Supplementary Methodology). Colour mapping indicates the TPP core (magenta) and cap domains (olive). The side chain of cysteine 222 is shown as a stick model. Figure prepared with PyMOL[24].

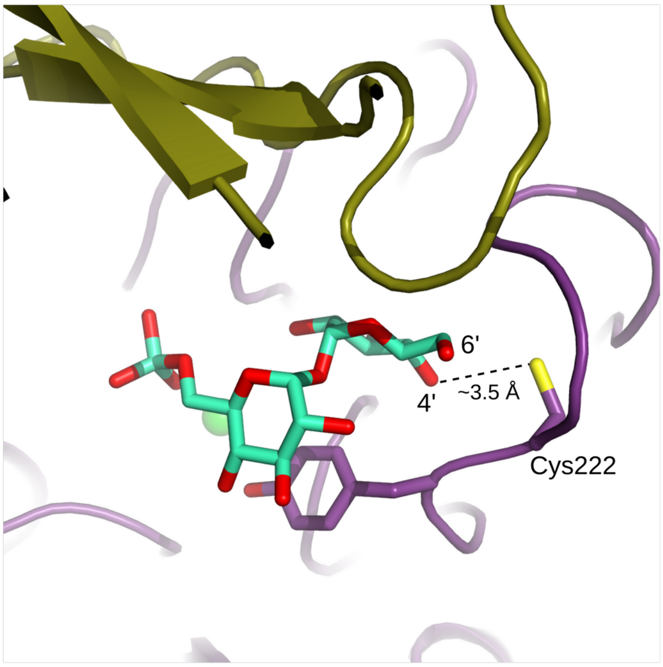

SUPPLEMENTARY FIGURE S3

Analysis of selected parameters from the molecular dynamics trajectory of *Bmal*-TPP:T6P

The plots of parameters versus simulation time indicate that the system was stable throughout the entire simulation and protein backbone conformation reached an equilibrium state. Stable binding of the substrate in the binding site is evident from the distance between the active site metal and the phosphorus atom of T6P.

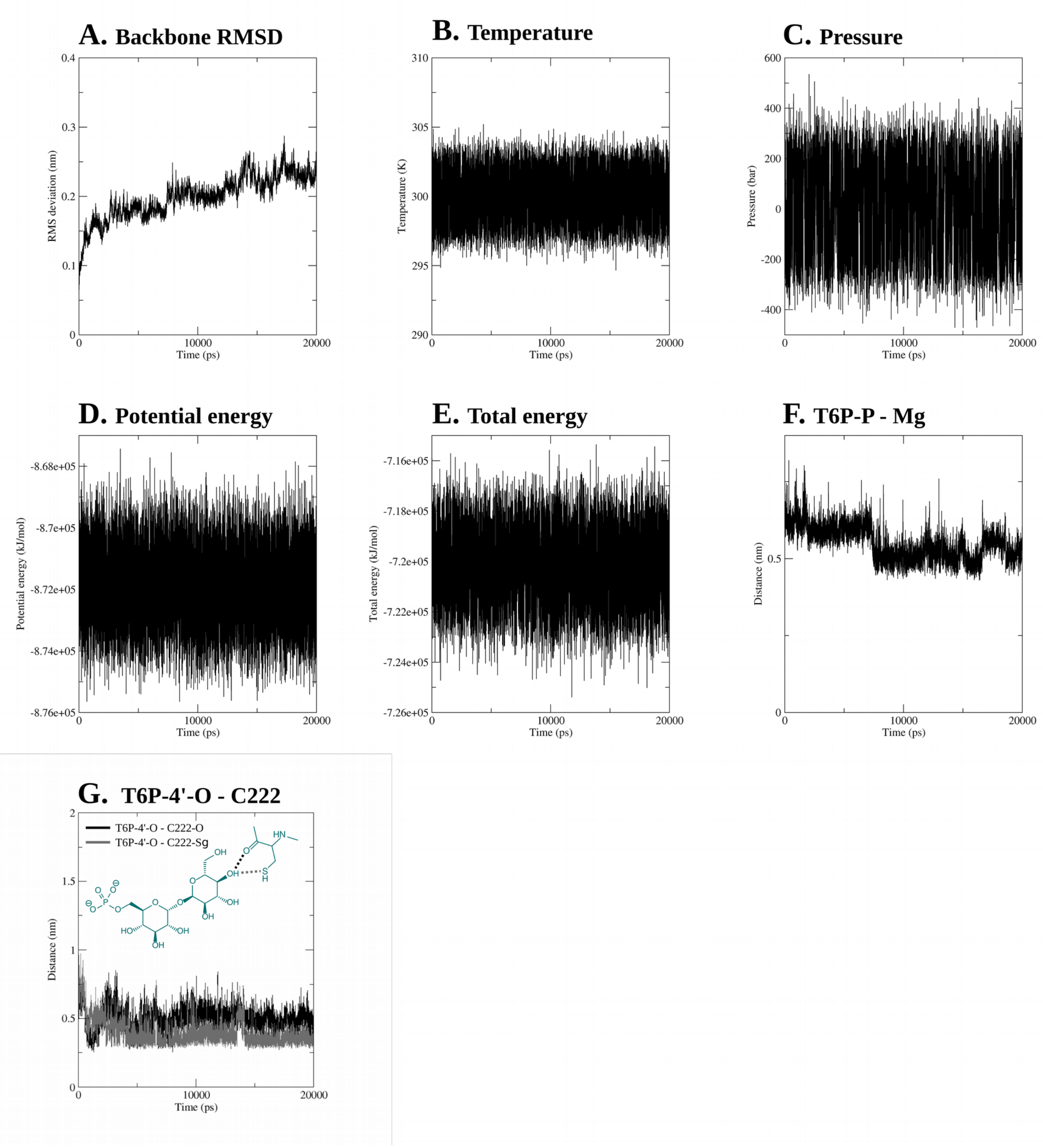

SUPPLEMENTARY FIGURE S4

Mass spectrometric analysis of *Tcan*-TPP incubated with *N*-(phenylthio)phthalimide confirms thiophenyl conjugation of the cysteine residues at positions 215 and 415

Peptides detected in the MS experiemnt are coloured in blue, unmatched peptides are shown in grey; cysteine residues are highlighted in yellow. Bold ‘C’ indicates thiophenyl conjugation and underlined ‘M’ indicates methionine oxidation.

1 MTVMAAESNR APKTKEDSRC ADEEHAHKED CVPQNAEKRP SEVSAAESGT
51 GSVNTIQTVD EFKALMYSMQ SVRRQIVAAI LSNNELENEW IETLNRTYAK
101 LTDSNTKAFQ REMSTISAKL SINIKDETTG LMKDLLYLDR LKAARSENRS
151 DSWPETLAKV DLISILAPYH PTSEQKFLEE FEGCLRFLRS FAESNTNGRK
201 PIFITDWDGT MKDY**C**SQYAT NLQPVYSAVG MTRFASRFTR LSAVLTAGPL
251 RGPGILDLTA MPIDGPVLFS GSWGREWWLG GRRVVHEDGI SDEGFDALQR
301 LNDEMSNLLH TGDYSQFALV GSGVQRKVDR LTLGVQTVYG HVLPELSHRY
351 QDAVKERMHR VDPQNHILVF DPSTELEVEV VAHNSGVVWN KADGVDRVVS
401 TVGDSLETPG KVLV**C**GDTHS DLPMVRQAVA RNPEGVMALF VGLNEKLRES
451 VRHLVGDQSR CCFVSCPDVI HAAMAELLNQ KRSTE

SUPPLEMENTARY FIGURE S5

Exposure to *N*-(phenylthio)phthalimide affects *Toxocara* L3 larvae survival and migration

Left panel: The survival of *T. canis* and *T. cati* larvae was assessed at different exposure times and varying concentrations of **1**. Circles represent the mean of six (*T. canis*) and four (*T. cati*) independent measurements; error bars indicate the standard error. Right panel: The amount of migrating *T. cati* larvae after 24 h exposure to varying concentrations of **1** was assessed in three (*T. canis*) and four (*T. cati*) independent experiments, respectively. Circles represent the mean and error bars indicate the standard error.

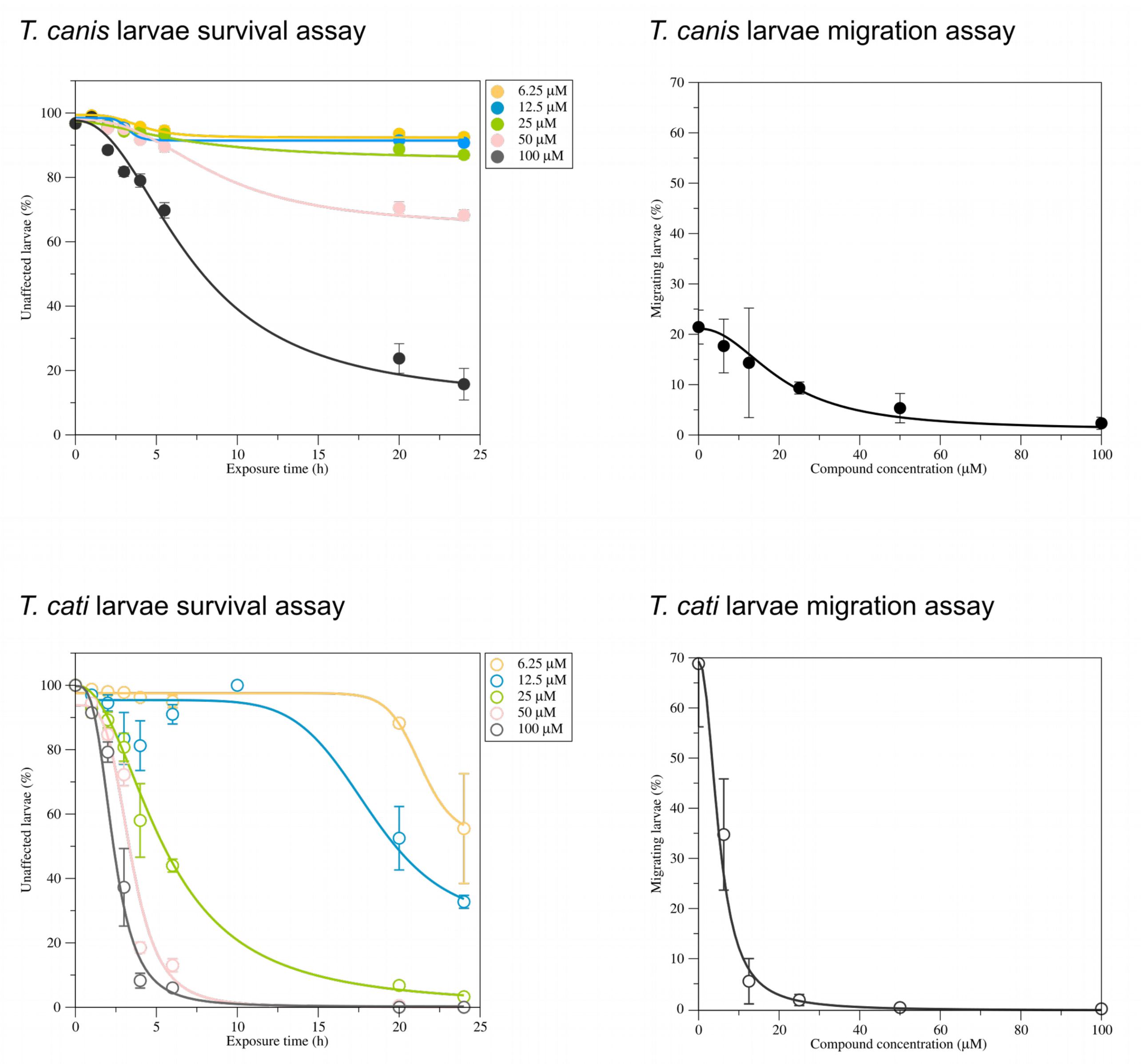

REFERENCES

1. Hofmann, A. *Methods in Structural Biochemistry*. (Structural Chemistry Program, Griffith University, 2018).
2. Petucci, C. & Diffendal, J. Atmospheric solids analysis probe: a rapid ionization technique for small molecule drugs. *J. Mass Spectrom.* **43**, 1565–1568 (2008).
3. Ray, A. D., Hammond, J. & Major, H. Molecular ions and protonated molecules observed in the atmospheric solids analysis probe analysis of steroids. *Eur. J. Mass Spectrom.* (2010). doi:10.1255/ejms.1069
4. van der Spoel, D. *et al.* GROMACS: Fast, flexible and free. *J Comp Chem* **26**, 1701–1718 (2005).
5. Schüttelkopf, A. W. & van Aalten, D. M. F. PRODRG: a tool for high-throughput crystallography of protein–ligand complexes. *Acta Crystallogr. D Biol. Crystallogr.* **60**, 1355–1363 (2004).
6. Darden, T., York, D. & Pedersen, L. Particle mesh Ewald: An N·log(N) method for Ewald sums in large systems. *J. Chem. Phys.* **98**, 10089–10092 (1993).
7. Bussi, G., Donadio, D. & Parrinello, M. Canonical sampling through velocity rescaling. *J. Chem. Phys.* **126**, 014101 (2007).
8. Parrinello, M. & Rahman, A. Polymorphic transitions in single crystals: A new molecular dynamics method. *J. Appl. Phys.* **52**, 7182–7190 (1981).
9. Hess, B., Bekker, H., Berendsen, H. J. C. & Fraaije, J. G. E. M. LINCS: A linear constraint solver for molecular simulations. *J. Comput. Chem.* **18**, 1463–1472 (1997).
10. Schwarz, E. M. *et al.* The genome and developmental transcriptome of the strongylid nematode *Haemonchus contortus*. *Genome Biol.* **14**, R89 (2013).
11. Preston, S. *et al.* Low cost whole-organism screening of compounds for anthelmintic activity. *Int. J. Parasitol.* **45**, 333–343 (2015).
12. Williams, A. R. *et al.* Anthelmintic activity of *trans*-cinnamaldehyde and A- and B-type proanthocyanidins derived from cinnamon (*Cinnamomum verum*). *Sci. Rep.* **5**, 14791 (2015).

13. Zhao, J. *et al.* An *in vitro* larval migration assay for assessing anthelmintic activity of different drug classes against *Ascaris suum*. *Vet. Parasitol.* **238**, 43–48 (2017).

14. Gillis, H. M., Greene, L. & Thompson, A. Preparation of sulfenyl pyrroles. *Synlett* **2009**, 112–116 (2009).

15. Hsieh, J.-C. & Cheng, C.-H. Nickel-catalyzed coupling of isocyanates with 1,3-iodoesters and halobenzenes: a novel method for the synthesis of imide and amide derivatives. *Chem. Commun.* 4554–4556 (2005). doi:10.1039/B506903C

16. Wang, Y. *et al.* PPh3/I2/HCOOH: An efficient CO source for the synthesis of phthalimides. *Tetrahedron* **75**, 1180–1185 (2019).

17. Heldreth, B. *et al.* N-Thiolated β-lactam antibacterials: Effects of the N-organothio substituent on anti-MRSA activity. *Bioorg. Med. Chem.* **14**, 3775–3784 (2006).

18. Zacharie, B. *et al.* Triazine derivatives, compositions containing such derivatives, and methods of treatment of cancer and autoimmune diseases using such derivatives (US Patent Application 2010/0129350). (2008).

19. Groutas, W. C. *et al.* Design, synthesis, and in vitro inhibitory activity toward human leukocyte elastase, cathepsin G, and proteinase 3 of saccharin-derived sulfones and congeners. *Bioorg. Med. Chem.* **4**, 1393–1400 (1996).

20. D'Ascenzio, M. *et al.* Design, synthesis and evaluation of N-substituted saccharin derivatives as selective inhibitors of tumor-associated carbonic anhydrase XII. *Bioorg. Med. Chem.* **22**, 1821–1831 (2014).

21. Wang, C. K. *et al.* SBAL: a practical tool to generate and edit structure-based amino acid sequence alignments. *Bioinformatics* **28**, 1026–1027 (2012).

22. Bryson, K. *et al.* Protein structure prediction servers at University College London. *Nucleic Acids Res.* **33**, W36–W38 (2005).

23. Cross, M. *et al.* Enzyme characteristics of pathogen-specific trehalose-6-phosphate phosphatases. *Sci. Rep.* **7**, 2015 (2017).

24. DeLano, W. L. The PyMOL Molecular Graphics System. *http://www.pymol.org* (2002).
